# Effect of Dog-Assisted Therapy on Psychomotor Development of Children with Intellectual Disability

**DOI:** 10.3390/children8010013

**Published:** 2020-12-29

**Authors:** Andżelina Wolan-Nieroda, Jadwiga Dudziak, Mariusz Drużbicki, Bogumiła Pniak, Agnieszka Guzik

**Affiliations:** 1 Department of Physiotherapy, Institute of Health Sciences, College of Medical Sciences, University of Rzeszów, 35-959 Rzeszow, Poland; dudziaku3653@wp.pl (J.D.); mdruzb@ur.edu.pl (M.D.); agnieszkadepa2@wp.pl (A.G.); 2 Spa and Rehabilitation Hospital “EXCELSIOR”, 38-440 Iwonicz Zdrój, Poland; gabipniak@vp.pl

**Keywords:** dog-assisted therapy, intellectual disability, psychomotor disorders, child developmental, kinesthesis, cognition disorders, dogs

## Abstract

Background: Although dog-assisted therapy (DAT) has been used for years, there is still a scarcity of research findings confirming efficacy of the method. The current study was designed to assess effects of DAT on psychomotor development of children with mild intellectual disabilities. Material and method: The study involved 60 children with mild intellectual disabilities, aged 10–13 years, divided into a group participating in a 10-month DAT program, and the control group. Four tests were applied, i.e., finger identification, postural imitation, kinaesthesia, and Bourdon-Wiersma Dot Cancellation Test. The examinations were carried out before the start and at the end of the DAT, and at a two-month follow-up. Results: The results obtained by the DAT group in all the four tests, at all the three timepoints, were not the same (*p* < 0.001). No statistically significant differences were found in the measurement at the end of the therapy between the DAT group and the controls. On the other hand, the DAT group achieved significantly better scores (*p* = 0.001 and *p* = 0.001), compared to the control, in the follow-up measurements two months after the end of the therapy in postural imitation and finger identification tests. Conclusions: Some of the scores achieved by the children in the DAT group improved in the measurements performed over time. Two months after the therapy ended, the children in the DAT group presented greater gains in motor planning (postural imitation test) and in the sense of touch, attention, and concentration (finger identification test), compared to the control group. Although the measurement performed immediately after the therapy did not show significant differences between the DAT group and the controls, the examination carried out at the two-month follow-up identified long-term gains in the treatment group in the domain of motor planning (postural imitation test).

## 1. Introduction

Positive effects of Animal Assisted Therapy (AAT) on various physical and mental characteristics have been reported worldwide in the last 20 years. More specifically, many studies suggest that Dog Assisted Therapy (DAT) produces positive results in children with various developmental disorders, contributing to their ability to concentrate and to feel motivated for work [1,2,3]. Furthermore, research investigating effectiveness of AAT in relation to patients’ social skills showed that interventions of this type favourably affected communication and social interactions between individuals with intellectual disability [3]. AAT is not only helpful as regards communication and basic activities of daily living. It has also been shown that in children with intellectual disability it may beneficially affect gross motor skills, since it can effectively boost a sense of motivation for exercise [4]. Dog-Assisted Therapy, also known as canine-assisted therapy or contact therapy, is gaining popularity and is well-tested as a form of complementary treatment [3,4,5]. Therapy dogs are used in rehabilitation of patients with motor and intellectual disabilities [5]. Contact therapy involving dogs is one of the methods that may be employed to promote the process of rehabilitation and recovery. Dogs are effectively used in treatment of individuals with disabilities or concentration and attention disorders because they favourably affect the psychophysical and socio-physical domains. Interaction with these animals reduces anxiety, simulates sense organs, increases vocabulary resources, and improves contact with the environment [6,7]. DAT enables children to improve gross motor skills, own-body perception as well as fine motor skills. It favourably affects cognitive skills, such as concentration, perceptivity, ability to take decisions, as well as ability to adequately perceive and respond to a given situation. Games and fun activities taking place during DAT facilitate learning and consolidation of such notions as colours, sizes, numbers, differences, and similarities. A review of the related literature shows that DAT also contributes to better psychomotor efficiency [8,9,10]. Studies by Jorge et al. showed positive effects of DAT in children’s motor development, particularly balance, motor planning, and spatial orientation [11]. It appears that DAT is particularly successful as a way to help individuals with intellectual disabilities and improve their psychomotor efficiency. Interaction with an animal leads to enhancement of neurotransmission in the human, which initiates decrease in blood pressure and induces relaxation. This association may be beneficial in reducing arousal as well as psychological symptoms of chronic diseases, including physical and mental disabilities [12,13]. A study by Scorzato et al., assessing effects of DAT in individuals with intellectual disability, showed significant improvement related to a number of cognitive factors, including focus on movement, visuomotor coordination, exploratory games, and imitation of motion. The effects of the therapy did not depend on the subjects’ age and degree of intellectual disability [14]. Gocheva et al. also reported statistically significant findings related to attention and concentration in patients with brain injury participating in DAT [15]. Kongable et al. observed positive effects of DAT in tactile and visual perception as well as verbalisation [13]. Systematic participation in appropriately structured activities involving a dog makes it possible for children using this type of therapy to improve their physical and mental condition. DAT promotes overall physical activity and motor capacities. Children with intellectual disability participating in exercise frequently have problems with motor activity, they are sluggish and unwilling to move. A dog motivates them to take action; they approach the animal to say hello, and they focus their attention on the dog. DAT improves precision of movements, as a result of which the child gains greater motor control [16]. This is also an excellent form of rehabilitation for a child. Presence of a dog helps in performance of self-care activities and reduces emotional tension. “Depending on the child’s needs, during dog-assisted therapy session the child may perform a number of exercises focusing on gross motor skills, manual efficiency and visual perception” [17]. Contact therapy involving a dog facilitates own-body schema orientation, allowing the child to understand the structure of both animal and human body, and to improve their motor efficiency, as a result of exercise performed together with the dog and through stimulation of the senses of vision, hearing, and touch as well as practice of attention and concentration. Numerous studies demonstrated effectiveness and usefulness of animals in therapy [12,13,14,15,16,17,18,19]. An animal does not only help to calm down and to develop one’s social behaviours but also constitutes a source of motivation [20]. Owing to their inherent qualities, animals may induce child’s interest and may stimulate a variety of sensory functions through sounds, movement, smell, and touch. Their activity is simple, repeatable, and non-verbal; as a result, it is more accessible even to individuals with language dysfunctions [21]. Animals are a source of and the purpose for attention [22]. Efforts to systematically categorise the ways humans are affected by animals made it possible to distinguish a few mechanisms:

- Affective—related to feelings aroused in a person by the animal;
- Psychological stimulation—interaction with the animal, stimulating social behaviours and cognitive functions;
- Recreational—related to activation of motor functions and motor planning [23,24].

Therapy intended for a child with intellectual disability should be intensive and multidimensional; it should boost the development of and strengthen the child’s intellectual functioning, as a result potentially increasing his/her independence. Because of this, therapy should be designed to stimulate perception-related functions of the brain, concentration, and attention, to improve motor functions, to promote development of speech and communication skills [20]. The methods facilitating rehabilitation of children with intellectual disability include DAG, which is intended to stimulate development in all the domains and promote improvement in motor capacities. The above review of literature suggests that DAT favourably affects concentration and attention [9], motor planning [15], spatial orientation [15] as well as sense of touch [21]. DAT is a widely used form of supplementary treatment. However, few reports so far have specifically focused on the effects of this type of intervention in children with intellectual disability [24,25]. Therefore, the present study aimed to investigate whether or not long-term/delayed effects of DAT would impact children’s performance during a period when they did not participate in education and rehabilitation programs.

In view of the above, the study was designed to assess psychomotor efficiency, reflected by the factors of attention and concentration, motor planning, spatial orientation, and sense of touch in children with mild intellectual disability participating in DAT and in controls receiving no such therapy, immediately after the period of education and rehabilitation and following a period during which the children did not receive DAT and did not participate in education and rehabilitation.

Hypotheses:

**Hypothesis 1**.  *Children with intellectual disability, participating in an educational program supplemented with DAT, achieve improvement in attention and concentration, motor planning, spatial orientation, and sense of touch, in assessments carried out at the end of the educational program and at a two-month follow up*.

**Hypothesis 2**.  *Improvement in the DAT study group is significantly greater than in the non-DAT control group*.

**Hypothesis 3**.  *Effects of education supplemented with DAT are long-lasting. Children additionally receiving DAT after a two-month break in the education program present greater improvement compared to the non-DAT control group*.

## 2. Materials and Methods

### 2.1. Participants

The study was conducted in a special educational facility in the Podkarpackie Region, Poland, and involved 60 children with mild intellectual disability, mean age 11 years ± 2.3 years. Eligibility criteria included: Mild intellectual disability (intelligence quotient of 50–70 according to Wechsler Intelligence Scale) [26,27], special school education, age 10–13 years, and parent’s/legal guardian’s consent to participate. The study protocol excluded children with moderate to severe intellectual disability with cognitive deficits impairing the ability to understand and follow instructions. Other exclusion criteria were defined as follows: Co-existing autism, cerebral palsy, total visual and hearing impairment, muscular dystrophy and neurological disorders such as brain injury and epilepsy. Prospective subjects were disqualified from the study if their parents/legal guardians failed to grant consent for participation.

### 2.2. Flow of the Subjects through the Study

**Figure 1:**
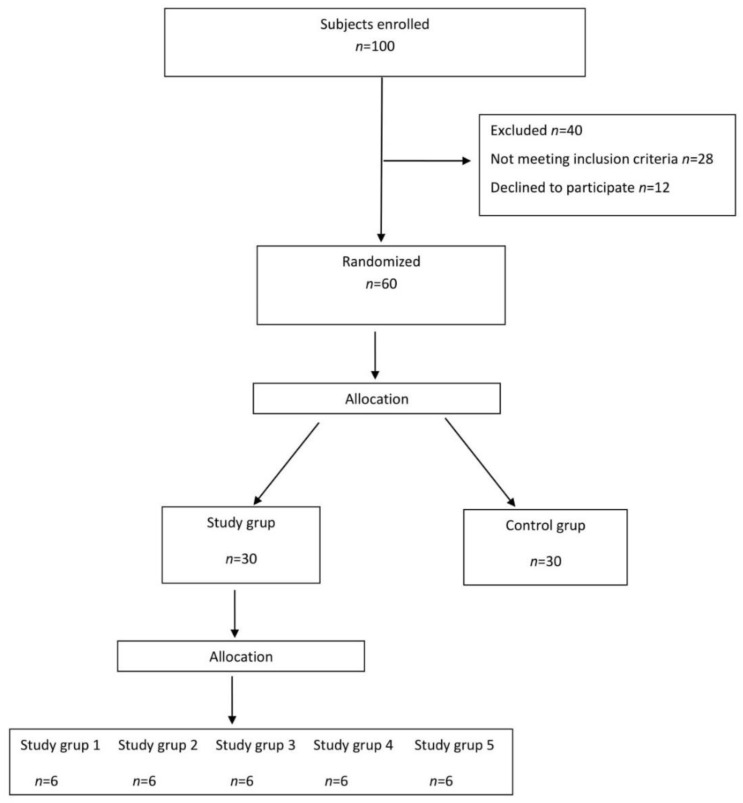
Flow of subjects through the study.

One hundred children were examined successively, as they were admitted to a special educational facility in the Podkarpackie Region, Poland. Ultimately 60 children were enrolled for the study. Out of the 40 children who were not qualified for the programme, 28 failed to meet the inclusion criteria, and 12 refused to participate. All the subjects completed the final examination. Figure 1 shows the flow of the subjects through the study.

### 2.3. Study Design

Single blind trial design was applied with randomised assignment of participants to one of the two parallel groups comprising a total of 60 children with intellectual disability, attending the same special educational facility in the Podkarpackie Region, Poland. Simple randomization was used, i.e., randomisation based on a single sequence of random assignments. The most common and basic method of simple randomisation was applied, which involved flipping a coin [28], the side of the coin (i.e., heads—DTA group, tails—control group) determined the assignment of each subject. First degree randomisation, i.e., random selection of subjects to two groups, the DTA group and the control group, whereby the children were allocated either to the group receiving DTA (the DTA group—30 children, 19 girls and 11 boys) or to the group that did not participate in the DAT program (the control group—30 children, 20 girls and 10 boys). Subsequently the DAT group was randomly divided by second degree randomisation, i.e., the subjects were assigned to five therapeutic subgroups, each comprising 6 children, relative to the procedures applied in the DAT. The subgroups had common characteristics in terms of functionality level (all the children presented the same functionality level in activities of daily living expressed by Barthel Index of 80–85 points), and cognitive level (mild intellectual disability—intelligence quotient of 50–70 according to Wechsler Intelligence Scale). All the children met all the inclusion criteria. The DAT program was continued for 10 months, with 45-min sessions taking place once a week.

### 2.4. Study Protocol

The protocol of this randomised study was approved by the local Bioethics Commission of the Medical Faculty (4 February 2017). The data presented in this article were obtained in a two-armed randomised controlled trial. Informed consent for the children’s participation in the study was obtained in writing from their parents or legal guardians. Experimental conditions were in compliance with the Declaration of Helsinki. No adverse events were observed during the study. Inclusion/exclusion criteria were met. No intentional deviations from the protocol were observed during the study.

### 2.5. Measurements

The assessments were carried out three times: At the start of the DAT program (Exam 1), at the end of the therapy program (i.e., after 10 months) (Exam 2), and at a two-month follow-up (Exam 3).

The following research tools were used:

- Bourdon–Wiersma Dot Cancellation Test—assessing concentration and attention. The subject is shown a sheet with a sequence of various letters and digits and is asked to quickly cross out specific letters, e.g., e and r, within 3 min. The result is based on the total number of characters crossed out correctly, and a total number of characters skipped and selected mistakenly. Each type of error may be indicative of an impairment in different processes of attention, e.g., weakening. A maximum of 102 points can be scored. In the entire sheet, the digit 6 appears 102 times. One point is scored for each digit 6 crossed out correctly [29].
- The Southern California Sensory Integration Tests (SCSIT Battery) proposed by J. Ayres, based on Southern California Sensory Integration Tests Manual, Los Angeles, Calif., Western Psychological Services [30,31]. The battery is designed to evaluate sensory integration by assessing e.g., kinaesthetic sense, perception of tactile stimuli, ability to visualise tactile sensations without visual control, ability to sense the location of tactile stimuli, as well as movement planning. The subtests used in the present study include postural imitation test—assessing motor planning and sequencing. A maximum of 12 points can be scored. The child is awarded 1 point if they correctly assume a position or 0 points if they fail to assume the position; finger identification test—assessing the sense of touch, attention and concentration. A maximum of 16 points can be scored, one point for each correct answer; as well as kinaesthesia test—assessing kinaesthetic sense, spatial positioning of extremities, and memory. A maximum of 24 points can be scored. The child is to indicate 12 locations where tactile stimuli were applied. Two points are scored for each correctly indicated location. If the child points to the correct part of the limb but not to the precise location, they score 1 point. If they fail to correctly indicate the location or the part of the limb, they score 0 points.

### 2.6. Procedure

The DAT program was continued for 10 months, with 45-min sessions held once a week. The sessions were carried out in groups of six children and aimed to improve functioning of memory and attention processes, to ensure adequate level of motivation, to increase sense of security and self-confidence in the presence of the dog, to boost the ability to cope with difficult emotions, to improve motor function and the sense of balance, and to reduce the sense of anxiety and loneliness through contact with the therapist and the dog. Some of the originally planned sessions had to be cancelled due to the patient’s or the therapist’s illness. All the DAT sessions were conducted in a therapy room in the premises of the facility. No therapy session had to be stopped earlier and no undesired events occurred. Needs of each patient and the goals of DAT (Table 1) were taken into account. The sessions in each of the five groups followed the same DAT program. In addition to that the children in the DAT group as well as the controls participated in a conventional treatment program, which included rehabilitation (individual practice focusing on endurance, correction, balance as well as strengthening of postural and respiratory muscles), speech therapy, as well as educational, artistic, and musical activities.

In our study, the DAT program was supervised by a team of experienced therapists for years providing services to children with intellectual disability.

### 2.7. Statistical Analysis

Statistical analyses were performed using Statistica 13.1. software developed by StatSoft Polska. The obtained results of the examinations did not meet the criteria for parametric tests, i.e., normality of distribution of the relevant variables, due to which alternative non-parametric tests were applied in the analyses. Compatibility of the distributions with normal distribution was verified using Shapiro–Wilk test. Comparison of the results achieved by the children in the DAT group in the consecutive timepoints relative to the therapy (measurement over time) was performed using Friedman’s ANOVA, Dunn’s test being a suitable post-hoc analytic tool. Comparison of the results within the two groups (DAT and control) was performed using Mann-Whitney U-test. Differences in the therapeutic effects (short- and long-term) in the same group of subjects were examined using paired samples Wilcoxon test. Statistical significance was assumed if *p* < 0.05.

### 2.8. Sample Size

The minimum size of the sample was calculated taking into account the number of children with intellectual disability attending the special educational facility in the Podkarpackie Region, Poland, annually. A fraction size of 0.9 was used, with a maximum error of 5% [32,33,34,35], a sample size of 58 children was obtained. The study involved 60 children. The following formula was applied to determine the minimum sample size:

$$N_{\min}\;=\frac{Np\left(\alpha^{2}\cdot f\left(1-f\right)\right)}{Np\cdot e^{2}+\;\alpha^{2}\cdot f\left(1-f\right)}$$

*N_min_*—minimum sample size
*N_P_*—size of the population sampled
*α*—confidence level for the results, value of Z-score in normal distribution for the assumed significance level, e.g., 1.96
*f*—fraction size
*e*—assumed maximum error expressed with a fractional number, e.g., 3% is expressed as 0.03

## 3. Results

Based on the examinations, statistical differences between the scores observed at the three timepoints—at the start of the DAT (Exam 1), at the end of the therapy program (i.e., after 10 months) (Exam 2), and at a two-month follow-up (Exam 3)—were assessed in the relevant group of children.

**Hypothesis 1**.  *Children with intellectual disability, participating in an educational program supplemented with DAT, achieve improvement in attention and concentration, motor planning, spatial orientation, and sense of touch, in assessments carried out at the end of the educational program and at a two-month follow up*.

It was shown that the results obtained by the DAT group in the category of finger identification, measured at the three timepoints (before the DAT, at the end of the DAT, and at a two-month follow-up) were not the same (*p* < 0.001). In order to identify statistically significant differences between the measurements at the specific timepoints, a post-hoc analysis was performed using Dunn’s test, appropriate for Friedman’s ANOVA. The analysis showed differences in the results between the measurements performed before and immediately after the therapy, measurements before the DAT and at the two-month follow-up, as well as measurements immediately after the DAT and at the two-month follow-up. Each subsequent measurement showed higher scores. Before the therapy the children achieved the lowest results; in the measurement immediately after the therapy there was a statistically significant increase and subsequently, at the two-month follow-up, again the scores were significantly higher relative to the short-term effect (Table 2).

It was shown that the results obtained by the DAT group in the category of postural imitation, measured at the three timepoints (before the DAT, at the end of the DAT, and at a two-month follow-up) were not the same (*p* < 0.001). The post hoc (Dunn’s) test showed there were differences between the results measured before DAT and at the two-month follow-up, as well as the results measured immediately after the DAT and at the two-month follow-up. The analysis did not confirm statistically significant differences between the results measured before the DAT and immediately after the DAT. The measurement before the DAT identified the lowest scores. Immediately after the DAT, the scores improved only slightly, however the further increase in the results, reflected by the difference in the measurements immediately after the DAT and at the two-month follow-up, was statistically significant. The result identified in the final measurement differed significantly from the baseline (Table 3).

It was shown that the results obtained by the DAT group in the category of kinaesthesia, measured at the three timepoints (before the DAT, at the end of the DAT, and at a two-month follow-up) were not the same (*p* < 0.001). The post hoc (Dunn’s) test showed there were differences between the results measured before DAT and immediately after the DAT, as well as the results measured before the DAT and at the two-month follow-up. The analysis did not confirm statistically significant differences between the results measured immediately after the DAT and at the two-month follow-up. The measurement before the DAT identified the lowest scores. Subsequently, there was a significant increase in the measurement immediately after the DAT and the effect was maintained, despite a small decrease, in the measurement at the two-month follow-up (Table 4).

It was shown that the results obtained by the DAT group in the Bourdon–Wiersma Dot Cancellation Test, measured at the three timepoints (before the DAT, at the end of the DAT, and at a two-month follow-up) were not the same (*p* < 0.001). The post hoc (Dunn’s) test showed there were differences between the results measured before DAT and immediately after the DAT, as well as the results measured before the DAT and at the two-month follow-up. The analysis did not confirm statistically significant differences between the results measured immediately after the DAT and at the two-month follow-up. The measurement before the DAT identified the lowest scores. Subsequently there was a significant increase in the measurement immediately after the DAT and the effect was maintained, but without a significant increase, in the measurement at the two-month follow-up (Table 5).

**Hypothesis 2**.  *Improvement in the DAT study group is significantly greater than in the non-DAT control group*.

The next part of the analyses involved comparison of the results achieved by the children in the DAT group at each stage of the therapy program to the scores of the children in the control group.

As regards finger identification the DAT group and the controls did not differ in the measurement before and immediately after the DAT, however the measurement two months after the DAT was completed showed statistically significant differences between the groups (*p* < 0.001), reflecting greater gains in the DAT group. The finding was confirmed by assessing effect size with Cohen’s d. Hence, the short-term changes in the two groups were comparable, however the performance of the DAT group reflected statistically better long-term effects possibly resulting from the therapy (Table 6).

No differences related to kinaesthesia were found between the DAT group and the controls in the measurements before and immediately after the DAT, and at the two-month follow-up. The finding was confirmed by assessing effect size with Cohen’s d (Table 7).

The scores in postural imitation test showed the DAT group and the controls did not differ in the measurements before and immediately after the DAT. On the other hand, the measurement at a two-month follow-up identified statistically significant differences (*p* < 0.001), with higher scores achieved by the DAT group. The finding was confirmed by assessing effect size with Cohen’s d. Hence, the short-term improvement in the two groups was comparable, however the scores of the DAT group seem to reflect statistically higher long-term effects of the therapy (Table 8).

As regards the Bourdon–Wiersma Dot Cancellation Test, the DAT group and the controls did not differ in the measurements at any stage of the therapy program. The finding was confirmed by assessing effect size with Cohen’s d (Table 9).

**Hypothesis 3**.  *Effects of education supplemented with DAT are long-lasting. Children additionally receiving DAT after a two-month break in the education program present greater improvement compared to the non-DAT control group*.

Comparative analysis examined relationships between measurement II and I (short-term effect—before the DAT versus immediately after the DAT) and measurement III and II (long-term effect—immediately after the DAT versus two-month follow-up).

As for the effect size identified in measurements I and II, as well as II and III, no significant differences were found in finger identification and in the Bourdon–Wiersma Dot Cancellation Test (*p* > 0.05). This means that immediately after DAT and at the two-month follow-up the children achieved similar results.

Assessment of the scores in postural imitation test showed that the long-term effect, reflected by measurement III versus II, was significantly greater than the short-term effect.

The related analyses of the scores in the kinaesthesia test showed a positive short-term effect and a negative long-term effect (*p* = 0.009), (Table 10). However, the previous findings (Table 3) showed that this decrease was not significant from the viewpoint of the therapy effectiveness because the final effect was similar to that observed immediately after the DAT and the scores were higher than those achieved before the DAT (Table 10).

## 4. Discussion

The present study investigated effects of DAT on psychomotor development of children with mild intellectual disability. Even though DAT has been used for years, the related gains from this type of intervention have rarely been assessed in children with intellectual disability [8,14]. Conversely, a quick review of the literature shows a number of publications assessing effects of DAT on the development of children with cerebral palsy, various motor disabilities, and autism [36,37,38].

The current study involved a group of children with mild intellectual disability, aged 10–13 years (mean age 11 years ± 2.3 years). The children presented poorer abilities reflected by scores in finger identification, postural imitation, and kinaesthesia tests, compared to healthy children in the same age group [30]. Bülent et al. assessed sensory integration and activities of daily living in children with developmental coordination disorder; the scores achieved by the study group in postural imitation tests were considerably lower, compared to the scores of their healthy peers. Similar results were obtained in localisation of tactile stimuli and kinaesthesia tests [39]. Schoemaker et al. reported similar findings in a study assessing perceptual skills in children with impaired coordination. Children from the study group achieved poorer scores in a test assessing tactile perception and attention [40].

The present study suggests that DAT contributes to improvement in concentration reflected by the scores in finger identification test and Bourdon–Wiersma Dot Cancellation test. Before the therapy, the children achieved the lowest results; in the measurement immediately after the therapy, there was a statistically significant increase. Likewise, François et al. in their publication discussed effects of DAT on the functioning of children with pervasive developmental disorders and reported that during the therapy the children focused mainly on the dog. When they were assisted by a therapy dog, they were able to concentrate better and they exhibited greater awareness of their social environment [39]. Reed et al. conducted a study focusing on DAT and its effectiveness in children with autism. Children with autism disorder participating in DAT were found with better ability to concentrate and focus their attention, had higher intellectual potential and ability to learn as well as decreased level of anxiety [41].

In accordance with the protocol of the current study, the DAT sessions were carried out in groups of six children, and aimed to improve functioning of memory and attention processes, to ensure adequate level of motivation, increase sense of security and self-confidence in the presence of the dog, boost the ability to cope with difficult emotions, improve motor function and the sense of balance, and reduce the sense of anxiety and loneliness through the contact with the therapist and the dog. The results achieved by the children in the DAT group in kinaesthetic sense, perception of tactile stimuli, ability to visualise tactile sensations without visual control, and ability to sense the location of tactile stimuli were significantly improved immediately after therapy. Nawrocka-Rohnka conducted a study involving children with cerebral palsy, autism, and intellectual disability. The DAT sessions were held in groups of three children. The greatest progress was observed in the ability to communicate commands (mean improvement by 37.89%). This result may reflect improved functioning related to both the intellectual domain—remembering the instruction and the sequence of gestures necessary in communicating commands—and the mental domain—development of the sense of self-efficacy and improved self-esteem. Another area of significant progress observed in that study was related to “expression on emotions” (mean improvement by 30.96%), corresponding to the socioemotional domain. The poorest effects were found in the area of “mobility” (mean improvement by 30.96%), corresponding to the motor domain [42].

In another study, Gee et al. investigated effects of DAT on motor efficiency of pre-schoolers with language disorders. The researchers assessed locomotion, balance, and coordination before and after DAT. The children achieved better scores when they were working in the presence of a dog [43]. In the current study, DAT sessions were designed to include practice of gross motor skills, balance, and motor coordination. The analysis did not confirm statistically significant differences between the results in motor planning measured before the DAT and immediately after the DAT. Importantly, however, the further improvement, reflected by the difference in the measurements immediately after the DAT and at the two-month follow-up, was statistically significant. The result identified in the final measurement at the two-month follow-up, differed significantly from the baseline. It is likely that this result is associated with the fact that the effects of DAT in the study group were strengthened by the children’s involvement in basic and complex activities of daily living in home setting during summer holidays. The learning process in fact may have required more time, and the summer break, which involved a change of the environment, enabled a consolidation of the skills acquired during the 10 months, since they had to be “brought home” from the setting of the day care centre. Other researchers also observed that assistance of a dog during therapy sessions is associated with patients’ greater motivation and confidence during therapeutic activities as well as improved motor skills following the therapy; the children are more independent and function more effectively in the daily life [44,45].

The current study compared effects of the therapy (short-term—at the end of a 10-month DAT and long-term—at the two-month follow-up). As for the effect size reflected by the relation between measurements I and II, as well as II and III, the long-term effect (measurement III versus II) was significantly greater than the short-term effect in the category of postural imitation while a reverse situation was observed in the results of kinaesthesia test. It can be assumed that the change identified at the end of the therapy program is directly associated with the effectiveness of DAT, while the significant change identified two months later may reflect the fact that during the therapy the children received a certain stimulus and learned new psychomotor skills, which required sufficient time to be further improved. Similar long-term effects of DAT were reported by Piek et al. who concluded that this type of therapy favourably affects development of motor functions in children. They examined 511 children and their results were compared after 6 months and then again after 18 months. They found a statistically significant difference (*p* = 0.035) in the improved motor skills in the children taking part in the DAT sessions [10].

A review of the effects of other therapies on the psychomotor development of children with intellectual disability suggests that Dog-Assisted Therapy evaluated in the present study leads to comparable positive outcomes. For example, Ferreira et al. analysed the effects of a psychomotor intervention from the perspective of sensorial integration in children with intellectual disability. The study group included children aged 5 to 12 years. The therapy was designed to include physical education, described as “psychomotor education/reeducation”, and comprised 44 sessions, 50 min each. The findings showed that the program affected the following psychomotor domains: Body schema, tonicity, laterality, as well as global and final praxis. Less visible effects were identified in balance and space-time structure [46]. Similar effects were identified in the current study. Furthermore, Lucas et al. carried out a systematic review with meta-analysis to examine effects of conservative therapies designed to improve gross motor skills in children with various neurodevelopmental disorders. Nine articles met inclusion criteria. The authors reported that some task-oriented interventions can effectively be used for the above purpose [47]. On the other hand, Wuang et al. in quasi-experimental controlled study investigated effectiveness of sensory integrative therapy, perceptual-motor approach, and neurodevelopmental treatment in children with mild intellectual disability. The three types of interventions were applied to 120 children randomly assigned to three specific subgroups. Assessments performed with measures of sensorimotor function carried out after the interventions showed significantly better scores in the treatment groups compared to the controls (receiving no treatment) on almost all measures. Sensory integrative therapy more visibly affected fine motor skills, upper-limb coordination, and sensory integration. Perceptual-motor therapy produced significant gains in the children’s gross motor skills, while the neurodevelopmental treatment resulted in the smallest changes in most measures taken into account [48]. Conversely, Bukhovets and Romanchuk assessed effects of Bobath therapy on psychomotor development in children aged 3–6 years, presenting with organic central nervous system involvement. The study was designed to evaluate the children’s psychophysical state before and after neurodevelopmental treatment continued for 10 days in hospital. The findings showed a positive dynamics of motor activity and motor skills learning, which confirms the effectiveness of Bobath therapy as a method supporting psychomotor development of children aged 3–6 years with organic lesions in the central nervous system [49]. These observations are not consistent with the findings reported by Zanon et al. who published a systematic review following the recommendations of the Cochrane Handbook for Systematic Reviews of Interventions to assess the effects of neurodevelopmental treatment (Bobath) for children with cerebral palsy. They performed a comprehensive search for clinical trials designed to assess Bobath method in comparison to conventional physical therapy applied to children with cerebral palsy and identified three randomized clinical trials involving 66 children. The analyses showed that effectiveness of neurodevelopmental treatment and conventional physical therapy did not differ in the case of gross motor function [50]. In summary, it may be concluded that sensorimotor approaches should be selected taking into account specific needs of a child because each approach may present advantages with regard to certain aspects of sensorimotor functions.

The current analyses did not identify between-group differences at the end of the therapy program (i.e., after 10 months), however the DAT Group achieved significantly better scores in postural imitation and finger identification tests, compared to the controls, at the follow-up exam two months after the therapy was completed, which may reflect delayed improvement attributable to the DAT. This may be associated with the fact that the follow-up exam was performed at the end of the summer holidays. Typically, during a break from the day care facility children spend more time at home where they do not receive any specialised therapy. In fact, we interviewed the parents to find out whether their children had participated in additional therapeutic activities in the summer. No parents reported that their children had received any form of therapy in that period. We cannot rule out, with absolute certainty, that some external factors indeed impacted the final results. It can, however, be speculated that the functions stimulated by the DAT therapy were more extensively (and freely) practiced in home setting which possibly provides more opportunities for a variety of independently performed activities, compared to a day care centre where activities are generally more structured. This may have led to the children’s increased confidence in the use of the newly acquired abilities. In other words, it could be suggested that the 10-month period of the DAT, which involved exposition of the children to the stimuli, was more of a preliminary (theoretical) training, while the summer break associated with a change of the environment provided room for independent practical training of the skills. It is possible this period of “extra practice” enabled the children from the DAT group to gain greater self-reliance in the activities of daily living, which was eventually reflected by better scores acquired during the follow-up tests, compared to the controls.

In summary, the brief literature review above shows that most studies report positive results; DAT favourably affects the patients subjected to such interventions. Hence, it may be postulated that DAT is a good way to promote the process of rehabilitation, which is also supported by the current findings. However, it is necessary to continue the related research in larger groups of subjects and to apply different research tools.

The strength of this study is linked with the fact that the analysis of the long-term effects of the therapy program is based on three assessments. Functional implications of this study are related to the fact that the acquired results may be of importance for clinical practice since they present evidence confirming long-term effectiveness of DAT and constitute an encouragement for introducing DAT as a supplementary method, which may be applied along with conventional therapies in children with mild intellectual disability in facilities providing treatment to this population.

### Study Limitations

The findings of the current study apply only to children with mild intellectual disability. It is necessary to continue research and investigate the effects of DAT related to psychomotor efficiency of children with moderate and severe intellectual disability. Another limitation of the study is linked with the low number of participants. In the case of small samples, there is a greater risk that the results may be unreliable; besides, in small samples, statistical significance is found less frequently. As a rule, it is assumed that the larger the sample, the easier it is to detect any changes. Further research should involve a larger group of children with intellectual disability, and it would also be worthwhile to investigate impact of other factors such as age and sex on effectiveness of DAT.

## 5. Conclusions

The study showed certain long-term effects of dog-assisted therapy in the functioning of children with mild intellectual disabilities, aged 10–13 years. The results achieved by the children in the DAT group in some cases were significantly improved, as reflected by measurements performed over time. There were no significant differences in the baseline results and in the tests performed at the end of the therapy program between the children in the DAT group and the controls. Ultimately, however, at the two-month follow-up, the DAT group achieved better results than the controls in motor planning (postural imitation test) and in the sense of touch, attention, and concentration (finger identification test). Despite the fact that the measurement performed at the end of the therapy did not show significant differences between the DAT group and the controls, the results acquired at the two-month follow-up reflected long-term gains in the treatment group in the domain of motor planning (postural imitation test). Our findings suggest that dog-assisted interventions may effectively be used as a complementary treatment in children with physical and mental disability. The acquired results may be of importance for clinical practice since they present evidence confirming long-term effectiveness of DAT and constitute an encouragement for introducing DAT as a supplementary method, which may be applied along with conventional therapies in children with mild intellectual disability in facilities providing treatment to this population. This fact should be taken into account by those designing therapeutic programs in facilities providing treatment to this population of children.

## Figures and Tables

**Table 1:** Description of Dog-Assisted Therapy (DAT) sessions.

Month/ Group 1–5	Week	Introductory Activities, Making Contact with the Dog, Grooming and Taking Care of the Dog	Practice of Gross Motor Skills, Balance and Motor Coordination	Practice of Fine Motor Skills	Exercises Involving Memory, Attention and Concentration	Exercises Stimulating Haptic Perception. Normalisation of Muscle Tone	Improvement of Body Schema and Spatial Orientation	Duration of DAT per Week
I	I	10	10	15		10		45
	II	10	10		15		10	45
	III	10	10	15		10		45
	IV	10	10		15		10	45
Total		40 min	40 min	30 min	30 min	20 min	20 min	
II	I	5	10	15		10	5	45
	II	5	10		15	10	5	45
	III	5	10	15		10	5	45
	IV	5	10		15	10	5	45
Total		20 min	40 min	30 min	30 min	40 min	20 min	
III	I	5	10		15	10	5	45
	II	5	10	15		10	5	45
	III	5	10		15	10	5	45
	IV	5	10	15		10	5	45
Total		20 min	40 min	30 min	30 min	40 min	20 min	
IV	I	5	10	15		10	5	45
	II	5	10		15	10	5	45
	III	5	10	15		10	5	45
	IV	5	10		15	10	5	45
Total		20 min	40 min	30 min	30	40 min	20 min	
V	I	5	10	15		10	5	45
	II	5	10		15	10	5	45
	III	5	10	15		10	5	45
	IV	5	10		15	10	5	45
Total		20 min	40 min	30 min	30	40 min	20 min	
VI	I	5	10	15		10	5	45
	II	5	10		15	10	5	45
	III	5	10	15		10	5	45
	IV	5	10		15	10	5	45
Total		20 min	40 min	30 min	30	40 min	20 min	
VII	I	5	10	15		10	5	45
	II	5	10		15	10	5	45
	III	5	10	15		10	5	45
	IV	5	10		15	10	5	45
Total		20 min	40 min	30 min	30	40 min	20 min	
VIII	I	5	10	15		10	5	45
	II	5	10		15	10	5	45
	III	5	10	15		10	5	45
	IV	5	10		15	10	5	45
Total		20 min	40 min	30 min	30 min	40 min	20 min	
IX	I	5	10	15		10	5	45
	II	5	10		15	10	5	45
	III	5	10	15		10	5	45
	IV	5	10		15	10	5	45
Total		20 min	40 min	30 min	30 min	40 min	20 min	
X	I	5	10	15		10	5	45
	II	5	10		15	10	5	45
	III	5	10	15		10	5	45
	IV	5	10		15	10	5	45
Total		20 min	40 min	30 min	30 min	40 min	20 min	
Total duration of DAT		3 h 40 min	6 h 40 min	5 h	5 h	6 h 20 min	3 h 20 min	

**Table 2:** Identification—analyses of the measurements performed over time.

Finger Identification	Basic Descriptive Statistics												
	Number	Mean	−95% CI	+95% CI		Median	Min.	Max.		First Quartile	Third Quartile	StDev	Effect Size
Before DAT-I	30	8.57	7.35	9.79		8.00	4.00	13.00		6.00	12.00	3.27	
Difference II-I	30	1.20	0.50	1.90		1.50	−6.00	4.00		0.00	2.00	1.86	0.47
Immediately after DAT-II	30	9.77	8.62	10.91		9.50	4.00	13.00		7.00	13.00	3.06	
Difference III-II	30	1.97	1.16	2.78		1.00	0.00	7.00		0.00	4.00	2.17	0.84
Two-month follow-up-III	30	11.73	11.13	12.34		12.00	8.00	13.00		11,.00	13.00	1.62	
Difference III-I	30	3.17	2.27	4.07		3.00	0.00	8.00		1.00	5.00	2.41	1.29
*p*	Chi^2 Friedman’s ANOVA (N = 30, df = 2) = 41.02326 *p* < 0.001 Absolute differences between rank sums are (approximately) significant if > 18.5436877917081 at a significance level = 0.05												
	Before DAT				Immediately after DAT				Two-month follow-up				
Before DAT	---				21				42				
Immediately after DAT	21				---				21				
Two-month follow-up	42				21				---				

CI: confidence interval.

**Table 3:** Postural imitation—analyses of the measurements performed over time.

Postural Imitation	Basic Descriptive Statistics												
	Number	Mean	−95% CI	+95% CI		Median	Min.	Max.		First Quartile	Third Quartile	StDev	Effect Size
Before DAT-I	30	7.57	6.32	8.81		8.50	3.00	11.00		4.00	11.00	3.34	
Difference II-I	30	0.83	0.51	1.16		1.00	0.00	3.00		0.00	1.00	0.87	0.26
Immediately after DAT-II	30	8.40	7.26	9.54		10.50	3.00	12.00		5.00	11.00	3.05	
Difference III-II	30	3.03	1.88	4.19		1.50	−3.00	11.00		1.00	5.00	3.09	1.29
Two-month follow-up-III	30	11.43	10.82	12.04		12.00	6.00	15.00		12.00	12.00	1.63	
Difference III-I	30	3.87	2.58	5.15		3.00	−3.00	12.00		1.00	6.00	3.44	1.55
*p*	Chi^2 Friedman’s ANOVA (N = 30, df = 2) = 46.85437 *p* < 0.001 Absolute differences between rank sums are (approximately) significant if > 18.5436877917081 at a significance level = 0.05												
	Before DAT				Immediately after DAT				Two-month follow-up				
Before DAT	---				17.5				48.5				
Immediately after DAT	17.5				---				31				
Two-month follow-up	48.5				31				---				

**Table 4:** Kinaesthesia—analyses of the measurements performed over time.

Kinaesthesia	Basic Descriptive Statistics												
	Number	Mean	−95% CI	+95% CI		Median	Min.	Max.		First Quartile	Third Quartile	StDev	Effect Size
Before DAT-I	30	15.07	12.54	17.60		17.00	1.00	24.00		8.00	20.00	6.77	
Difference II-I	30	1.70	1.07	2.33		1.00	0.00	6.00		0.00	3.00	1.68	0.25
Immediately after DAT-II	30	16.77	14.19	19.34		19.00	2.00	24.00		11.00	24.00	6.90	
Difference III-II	30	−0.50	−2.07	1.07		0.00	−12.00	8.00		−3.00	2.00	4.22	0.08
Two-month follow-up-III	30	16.27	14.09	18.44		17.50	4.00	24.00		12.00	21.00	5.82	
Difference III-I	30	1.20	−0.48	2.88		2.00	−12.00	9.00		0.00	4.00	4.51	0.19
*p*	Chi^2 Friedman’s ANOVA (N = 30, df = 2) = 19.38947 *p* < 0.001 Absolute differences between rank sums are (approximately) significant if > 18.5436877917081 at a significance level = 0.05												
	Before DAT				Immediately after DAT				Two-month follow-up				
Before DAT	---				27				25.5				
Immediately after DAT	27				---				1.5				
Two-month follow-up	25.5				1.5				---				

**Table 5:** Bourdon–Wiersma Dot Cancellation—analyses of the measurements performed over time.

Bourdon-Wiersma Dot Cancellation Test	Basic Descriptive Statistics												
	Number	Mean	−95% CI	+95% CI		Median	Min.	Max.		First Quartile	Third Quartile	StDev	Effect Size
Before DAT-I	30	25.70	18.41	32.99		19.50	2.00	54.00		8.00	52.00	19.52	
Difference II-I	30	3.30	2.21	4.39		3.50	−1.00	10.00		1.00	5.00	2.91	0.16
Immediately after DAT-II	30	29.00	21.20	36.80		24.00	2.00	60.00		9.00	54.00	20.90	
Difference III-II	30	4.50	−1.59	10.59		5.50	−54.00	45.00		0.00	8.00	16.32	0.21
Two-month follow-up-III	30	33.50	25.56	41.44		33.00	5.00	60.00		10.00	59.00	21.26	
Difference III-I	30	7.80	1.85	13.75		8.00	−47.00	50.00		2.00	12.00	15.94	0.38
*p*	Chi^2 Friedman’s ANOVA (N = 30, df = 2) = 29.05556 *p* < 0.001 Absolute differences between rank sums are (approximately) significant if > 18.5436877917081 at a significance level = 0.05												
	Before DAT				Immediately after DAT				Two-month follow-up				
Before DAT	---				25.5				39				
Immediately after DAT	25.5				---				13.5				
Two-month follow-up	39				13.5				---				

**Table 6:** Finger identification—comparison of the scores achieved by the DAT group and the controls.

Finger Identification	Basic Descriptive Statistics													
	Number	Mean	−95% CI	+95% CI	Median	Min.	Max.	First Quartile	Third Quartile	StDev	Cohen’s d	Mann-Whitney U-Test–Z	*p*	Effect Size
DAT group—before therapy	30	8.57	7.35	9.79	8.00	4.00	13.00	6.00	12.00	3.27	0.02	0.04	0.971	0.02
Control group	30	8.50	7.53	9.47	8.00	4.00	13.00	6.00	11.00	2.60				
Immediately after DAT	30	9.40	8.49	10.31	10.00	4.00	14.00	8.00	11.00	2.43	0.04	0.57	0.564	0.04
Control group	30	9.50	8.49	10.51	9.50	5.00	15.00	8.00	12.00	2.70				
Two-month follow-up	30	11.73	11.13	12.34	12.00	8.00	13.00	11.00	13.00	1.62	1.15	3.89	<0.001	1.15
Control group	30	9.40	8.49	10.31	10.00	4.00	14.00	8.00	11.00	2.43				

**Table 7:** Kinaesthesia—comparison of DAT group scores achieved over time to the results of the controls.

Kinaesthesia	Basic Descriptive Statistics													
	Number	Mean	−95% CI	+95% CI	Median	Min.	Max.	First Quartile	Third Quartile	StDev	Cohen’s d	Mann-Whitney U-Test–Z	*p*	Effect Size
DAT group—before therapy	30	15.07	12.54	17.60	17.00	1.00	24.00	8.00	20.00	6.77	0.30	1.18	0.240	0.30
Control group	30	13.23	12.00	6.00	24.00	8.00	17.00	5.35	17.00	5.35				
Immediately after DAT	30	16.77	14.19	19.34	19.00	2.00	24.00	11.00	24.00	6.90	0.40	1.63	0.104	0.40
Control group	30	14.30	12.25	16.35	14.00	6.00	25.00	10.00	18.00	5.48				
Two-month follow-up	30	16.27	14.09	18.44	17.50	4.00	24.00	12.00	21.00	5.82	0.41	1.60	0.110	0.41
Control group	30	14.00	12.04	15.96	13.50	5.00	25.00	10.00	17.00	5.26				

**Table 8:** Postural imitation—comparison of DAT group scores achieved over time to the results of the controls.

Postural Imitation	Basic Descriptive Statistics													
	Number	Mean	−95% CI	+95% CI	Median	Min.	Max.	First Quartile	Third Quartile	StDev	Cohen’s d	Mann-Whitney U-Test–Z	*p*	Effect Size
DAT group—before therapy	30	7.57	6.32	8.81	8.50	3.00	11.00	4.00	11.00	3.34	0.35	−0.58	0.559	0.35
Control group	30	8.47	7.77	9.17	8.00	4.00	12.00	7.00	10.00	1.87				
Immediately after DAT	30	8.40	7.26	9.54	10.50	3.00	12.00	5.00	11.00	3.05	0.50	−0.86	0.387	0.51
Control group	30	9.80	8.88	10.72	10.00	5.00	15.00	9.00	11.00	2.46				
Two-month follow-up	30	11.43	10.82	12.04	12.00	6.00	15.00	12.00	12.00	1.63	0.94	3.78	<0.001	0.94
Control group	30	9.37	8.34	10.39	9.00	4.00	15.00	8.00	11.00	2.75				

**Table 9:** Bourdon–Wiersma Dot Cancellation Test—comparison of DAT group scores achieved over time to the results of the controls.

Bourdon-Wiersma Dot Cancellation Test	Basic Descriptive Statistics													
	Number	Mean	−95% CI	+95% CI	Median	Min.	Max.	First Quartile	Third Quartile	StDev	Cohen’s d	Mann-Whitney U-Test–Z	*p*	Effect Size
DAT group—before therapy	30	25.70	18.41	32.99	19.50	2.00	54.00	8.00	52.00	19.52	0.12	−1.03	0.301	0.12
Control group	30	27.67	23.01	32.32	23.50	6.00	54.00	19.00	36.00	12.47				
Immediately after DAT	30	29.00	21.20	36.80	24.00	2.00	60.00	9.00	54.00	20.90	0.00	−0.40	0.690	0.00
Control group	30	28.93	24.33	33.53	26.00	7.00	54.00	20.00	37.00	12.32				
Two-month follow-up	30	33.50	25.56	41.44	33.00	5.00	60.00	10.00	59.00	21.26	0.31	0.79	0.429	0.31
Control group	30	28.17	23.42	32.91	26.00	7.00	54.00	19.00	36.00	12.71				

**Table 10:** Comparison of the therapy effects, short-term and long-term.

		Basic Descriptive Statistics													
		Number	Mean	−95% CI	+95% CI	Median	Min.	Max.	First Quartile	Third Quartile	StDev	Cohen’s d	Paired Samples Wilcoxon Test–Z	*p*	Effect Size
Finger identification	Difference II-I	30	1.20	0.50	1.90	1.50	−6.00	4.00	0.00	2.00	1.86	−0.38	0.99	0.322	0.38
	Difference III-II	30	1.97	1.16	2.78	1.00	0.00	7.00	0.00	4.00	2.17				
Kinaesthesia	Difference II-I	30	1.70	1.07	2.33	1.00	0.00	6.00	0.00	3.00	1.68	0.75	2.60	0.009	0.75
	Difference III-II	30	−0.50	−2.07	1.07	0.00	−12.00	8.00	−3.00	2.00	4.22				
Postural imitation	Difference II-I	30	0.83	0.51	1.16	1.00	0.00	3.00	0.00	1.00	0.87	1.11	3.41	0.001	1.11
	Difference III-II	30	3.03	1.88	4.19	1.50	−3.00	11.00	1.00	5.00	3.09				
Bourdon- Wierman Test	Difference II-I	30	3.30	2.21	4.39	3.50	−1.00	10.00	1.00	5.00	2.91	0.12	0.84	0.400	0.12
	Difference III-II	30	4.50	−1.59	10.59	5.50	−54.00	45.00	0.00	8.00	16.32				

## Data Availability

Data available in a publicly accessible repository.
